# Rare Testicular Tumor Discovered by Assault: An Unusual Presentation of a Primary Testicular Neuroendocrine Tumor Grade 2

**DOI:** 10.1155/2013/709352

**Published:** 2013-09-19

**Authors:** Jonathan R. Epperson, Necia M. Pope, Margaret J. Abuzeid

**Affiliations:** ^1^Department of Pathology and Laboratory Services, San Antonio Military Medical Center, 3551 Roger Brooke Dr, Fort Sam Houston, San Antonio, TX 78234, USA; ^2^Department of Urology, San Antonio Military Medical Center, 3551 Roger Brooke Dr, Fort Sam Houston, San Antonio, TX 78234, USA

## Abstract

Testicular neuroendocrine tumors (NET) or carcinoid tumors are rare neoplasms which represent 1% of all testicular tumors and can be divided into 3 subgroups: pure primary testicular NET, primary testicular NET associated with a teratoma, and NET metastases to the testis. We report an unusual presentation of a primary testicular neuroendocrine tumor in a 39-year-old male who presented after a physical altercation during a soccer game. Histology showed a diffuse infiltrating tumor with extensive involvement of the tunica albuginea and tunica vaginalis. Immunohistochemical expression of CD56, synaptophysin, and chromogranin A was strongly positive in the tumor cells. Foci of tumor cell necrosis and occasional mitotic figures as well as extensive lymph-vascular invasion were also identified. A review of the literature reveals differing opinions on the prognostic significance of primary tumor size, mitotic index, tumor necrosis, and nuclear atypia. In our patient, the increased mitotic rate (3–5 mitotic figures per 10 hpf and a Ki-67 index of 5%), foci of necrosis, and mild to moderate nuclear atypia warranted a diagnosis of neuroendocrine tumor grade 2, formerly atypical carcinoid. Long term surveillance in these patients is essential as metastasis occurs in up to 15% of cases. At the 6-month followup, the patient remains symptom free.

## 1. Introduction

Testicular neuroendocrine tumors (carcinoid tumors) are rare neoplasms which represent 1% of all testicular tumors [1] and may behave in an either indolent or aggressive course [2]. We report an unusual presentation of a primary testicular neuroendocrine tumor in a 39-year-old male who came into the emergency department with left scrotal enlargement after a physical altercation during a soccer game where he was repeatedly kicked in the face, abdomen, and groin.

## 2. Case Presentation

**Figure 1:**
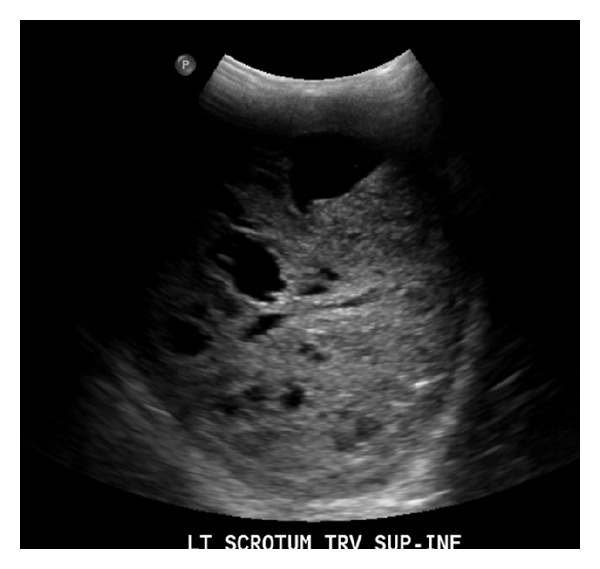
Ultrasound of left testis showing hypoechoic enlargement and scattered lobulated anechoic cystic foci of various sizes.

Upon presentation to the emergency room, the patient reported multiple blows to the face and eye. Initial evaluation preoperatively revealed an eyelid laceration for which the patient was taken urgently to the OR for repair. Intraoperative exam revealed a previously unnoticed massively enlarged left hemiscrotum without ecchymosis. Upon further questioning of the patient's wife regarding the history, it was noted that the patient had an at least 15-year history of asymptomatic enlarged scrotum on the left side which had not been previously evaluated. Ultrasound showed a diffuse, heterogeneous, and predominately hypoechoic enlargement of both testicles without increased vasculature and scattered lobulated anechoic cystic foci of various sizes, as well as an irregular contour in the left testicle (Figure 1). The ultrasound differential included testicular rupture from trauma or possible malignancy. Serum tumor markers human chorionic gonadotrophin (HCG beta subunit), alpha fetoprotein (AFP), and lactate dehydrogenase (LDH) were negative.

Intraoperative bilateral scrotal exploration was performed. There was a small hydrocele in the right hemiscrotum, and inspection of the right testicle was unremarkable. The left hemiscrotum contained a large left hematocele which was drained, revealing a markedly enlarged left testicle with a thickened, firm tunica albuginea containing multiple irregular nodules. A left orchiectomy was performed due to the suspicious operative findings. Gross examination revealed several small firm nodules on the tunica albuginea; the largest had heaped borders and measured 4.2 cm in greatest dimension. Sections of the testicle showed a heterogeneous yellow tan mass infiltrating the testicular parenchyma. Also, within the testicular parenchyma was a multiloculated hemorrhagic cystic nodule measuring approximately 3.2 cm. The remaining testicular parenchyma was distorted, congested, and edematous with a large, gelatinous hemorrhagic clot. 

**Figure 2:**
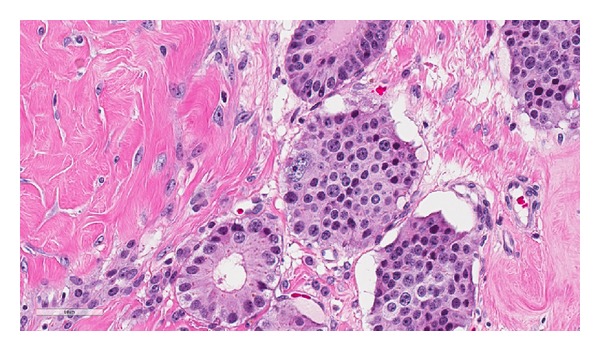
Tumor cells arranged in a nested and pseudoglandular pattern with abundant eosinophilic cytoplasm and coarse granular chromatin. An atypical cell is seen in the center (H&E, 40x).

**Figure 3:**
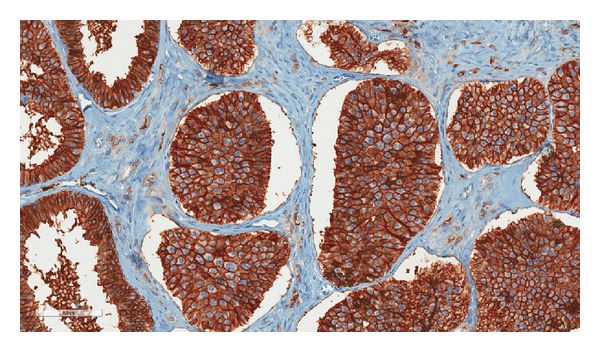
Tumor cells show strong membranous and cytoplasmic expression of CD56. The tumor cells also expressed synaptophysin and chromogranin A facilitating a diagnosis of neuroendocrine tumor. Original magnification 20x.

**Figure 4:**
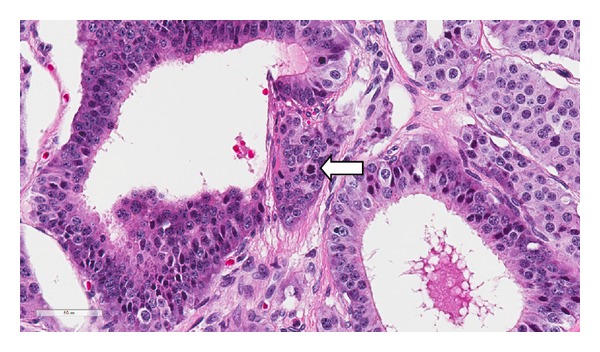
A mitotic figure is seen (arrow) in this 40x field. The patient had 3–5 mitotic figures per 10 hpf (H&E, 40x).

**Figure 5:**
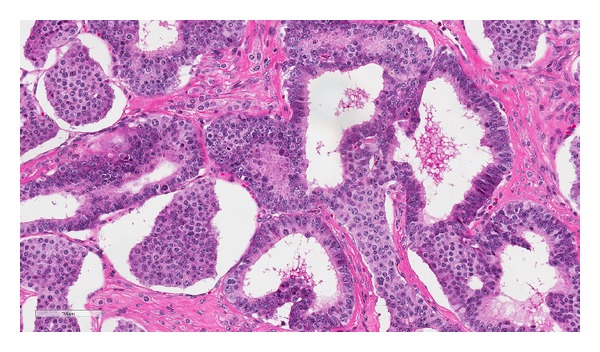
Foci of tumor necrosis and scattered apoptotic bodies are seen warranting a diagnosis of neuroendocrine tumor grade 2 (H&E, 40x).

 Histology showed a diffuse infiltrating tumor with extensive involvement of the tunica albuginea and tunica vaginalis. The tumor was composed of moderately pleomorphic cells in a pseudoglandular and nested pattern. The tumor cell nuclei had coarsely granular chromatin, surrounded by abundant eosinophilic cytoplasm (Figure 2). Immunohistochemical expression of CD56, synaptophysin, and chromogranin A was strongly positive in the tumor cells, while CD117, CD30, epithelial membrane antigen (EMA), alpha-inhibin, and Oct-4 nuclear stain were negative, consistent with a neuroendocrine tumor (Figure 3). Foci of tumor cell necrosis and occasional mitotic figures (>2 per 10 hpf) as well as extensive lymph-vascular invasion were also identified (Figures 4 and 5). No teratomatous elements or intratubular germ cell neoplasia was identified. The remaining testicular parenchyma was hemorrhagic and edematous with little residual normal testis. 

Clinically, the patient denied all symptoms and signs of a carcinoid syndrome. Urinary 5-hydroxyindoleacetic acid levels were not elevated, 2.4 mg/L and 7.6 mg/24 hour. However, chromogranin A was moderately elevated at 9 nmol/L. Postoperative CT of the chest, abdomen, and pelvis was normal except for borderline enlarged retroperitoneal lymph nodes on the left side, which measured up to 1 cm. The tumor was diagnosed as a primary neuroendocrine tumor grade 2 (G2) of the testis.

At the 6-month followup, the patient remains symptom free. 5-HIAA 24 hr urine, CBC, CEA, CA 19–9, and chromogranin A levels were all normal. Scrotal ultrasound demonstrated a normal right testis. Repeat abdomen and pelvis CT showed stable lymphadenopathy with no signs of recurrence. 

## 3. Discussion

Testicular neuroendocrine tumors (NET) or carcinoid tumors of the testis are rare, comprising less than 1% of all testicular tumors, and they can be divided into 3 sub-groups: pure primary testicular NET, primary testicular NET associated with a teratoma, and NET metastases to the testis [1, 3]. Pure primary testicular NET accounts for the majority of reported cases, followed by NET associated with a teratoma and metastasis to the testis accounting for the least number of cases [2–4]. They occur in a slightly older group of patients compared to germ cell tumors with a mean and median age at presentation of 46 years with a range of 10–83 years of age. Patients commonly present with painless testicular enlargement or a discrete testicular mass and rarely manifest with symptoms of carcinoid syndrome, although elevated levels of serotonin may be present [1]. This is in contrast to neuroendocrine tumors arising in the ovaries, in which it is more common for them to be associated with a teratoma. Also, since the ovarian blood supply drains directly into the vena cava and does not need to metastasize to the liver to cause a carcinoid syndrome, up to one-third of women with an ovarian NET may have an associated carcinoid syndrome [5].

While it is noted that the prognosis of testicular NET arising within a teratoma is better than pure testicular NET [2], determining factors that would predict prognosis in pure testicular neuroendocrine tumors has been limited due to the rarity of cases; however, in a recent series of 10 cases by Reyes et al., it has been shown that when testicular neuroendocrine tumors are graded in a three-tiered system (low grade or G1, intermediate grade or G2, and high grade or G3) using the criteria for lung neuroendocrine tumors, that is, mitotic rate, degree of nuclear atypia, and presence or absence of necrosis, patients with intermediate grade (G2) testicular neuroendocrine tumors have worse prognoses [6]. On the other hand, in an older review of 66 cases by Zavala-Pompa et al., tumor necrosis, mitotic activity, and vascular or tunica albuginea invasion appeared to have no effect on the behavior of this neoplasm, whereas large tumor size and the presence of carcinoid syndrome resulted in a greater likelihood of metastatic disease [2]. The term neuroendocrine carcinoma as presented by Reyes for all primary testicular neuroendocrine neoplasms (grades 1, 2, and 3) may better represent the potential for aggressive disease and is preferred over carcinoid tumor. In our patient, the slightly increased mitotic rate (3–5 mitotic figures per 10 HPF and a Ki-67 index of 5%), foci of necrosis, and mild to moderate nuclear atypia warranted a diagnosis of intermediate-grade (G2) neuroendocrine tumor or carcinoma. 

The management of localized well-differentiated testicular neuroendocrine tumor is complete surgical excision. The role of adjuvant treatment such as conventional chemotherapy or radiotherapy for higher grade tumors is not well defined. Somatostatin analogues have shown to be effective in improving prognosis [7]. 

In our patient, initial chromogranin A was slightly elevated, but follow-up levels were normal. Plasma levels of chromogranin A have been used as a marker for recurrence in patients with midgut neuroendocrine tumors with fairly good sensitivity [8]. Markedly elevated levels of chromogranin A (>5000 micrograms/L) are associated with a worse outcome in gastrointestinal NET [9]. However, the significance of elevated serum chromogranin A in testicular NET has yet to be determined. 

Long term surveillance and followup in these patients are essential as metastasis occurs in up to 15% of cases, and late disease recurrence has been reported many years after orchiectomy, with at least one reported case in the literature of a recurrence after 17 years [10, 11]. The use of both abdominal CT and somatostatin receptor scintigraphy (SRS) for monitoring the recurrence and metastasis is helpful [12]. Some investigations have found 24-hour urine collection for 5-HIAA useful for monitoring disease recurrence [12–14].
